# The Potential of Accelerating Early Detection of Autism through Content Analysis of YouTube Videos

**DOI:** 10.1371/journal.pone.0093533

**Published:** 2014-04-16

**Authors:** Vincent A. Fusaro, Jena Daniels, Marlena Duda, Todd F. DeLuca, Olivia D’Angelo, Jenna Tamburello, James Maniscalco, Dennis P. Wall

**Affiliations:** 1 Center for Biomedical Informatics, Harvard Medical School, Boston, Massachusetts, United States of America; 2 Department of Pathology, Beth Israel Deaconess Medical Center, Boston, Massachusetts, United States of America; 3 Department of Pediatrics, Division of Systems Medicine, Stanford University, Stanford, California, United States of America; California Department of Public Health, United States of America

## Abstract

Autism is on the rise, with 1 in 88 children receiving a diagnosis in the United States, yet the process for diagnosis remains cumbersome and time consuming. Research has shown that home videos of children can help increase the accuracy of diagnosis. However the use of videos in the diagnostic process is uncommon. In the present study, we assessed the feasibility of applying a gold-standard diagnostic instrument to brief and unstructured home videos and tested whether video analysis can enable more rapid detection of the core features of autism outside of clinical environments. We collected 100 public videos from YouTube of children ages 1–15 with either a self-reported diagnosis of an ASD (N = 45) or not (N = 55). Four non-clinical raters independently scored all videos using one of the most widely adopted tools for behavioral diagnosis of autism, the Autism Diagnostic Observation Schedule-Generic (ADOS). The classification accuracy was 96.8%, with 94.1% sensitivity and 100% specificity, the inter-rater correlation for the behavioral domains on the ADOS was 0.88, and the diagnoses matched a trained clinician in all but 3 of 22 randomly selected video cases. Despite the diversity of videos and non-clinical raters, our results indicate that it is possible to achieve high classification accuracy, sensitivity, and specificity as well as clinically acceptable inter-rater reliability with nonclinical personnel. Our results also demonstrate the potential for video-based detection of autism in short, unstructured home videos and further suggests that at least a percentage of the effort associated with detection and monitoring of autism may be mobilized and moved outside of traditional clinical environments.

## Background

In the United States, autism spectrum disorder (ASD) is typically diagnosed in children at four years of age with an estimated 27% undiagnosed by eight years of age [1]. Yet, the majority of parents of ASD children report developmental concerns before one year of age [1]–[3]. To better understand the developmental progression of ASD and identify distinguishing behaviors between ASD and non-ASD children, a number of studies focused on retrospective home video analysis [4]–[12]. Previous home video studies showed that ASD and non-ASD children differ in frequency of responding to name [10], gaze [13], smiling [14], and stereotypic motor behaviors [15]. It is important to note that home videos generally will not reflect all aspects of the clinical evaluation and can present challenges to proper diagnosis such as not clearly capturing eye contact, capturing context-dependent behaviors that may not exemplify routine, and poorly demonstrating social behavior with unfamiliar person(s). Nevertheless, home videos are considered a more accurate representation of early events than parental recall [10], and could be of value in future efforts focused on lowering the average age of diagnosis in the Unites States and abroad.

Significant prior work has focused on creating structured questions and standardized approaches to evaluate home videos by retrospectively examining early videos of children later diagnosed with ASD. This allows researchers to combine additional clinical features, such as age and IQ, with the video to develop a more complete representation of behavioral development. To standardize the home videos, most research focuses on a common event such as first or second birthday, and/or pivotal developmental milestones (such as not pointing, not responding to name calling, or not playing [stereotypically or pretend]).

We sought to expand the concept and feasibility of home video analysis by applying the Autism Diagnostic Observation Schedule (ADOS) [16] questions, not the full exam, to ASD and non-ASD videos from YouTube, the largest public video-sharing Internet website. The ADOS is widely considered a gold standard and is one of the most common behavioral instruments used to aid in diagnosis of ASD [16]–[18]. Clinical application of the ADOS requires a clinician trained in administrating the exam in order to elicit specific types of responses during structured activities. The exam is divided into four modules that correspond to an individual’s language and developmental level, ensuring coverage for a wide variety of behavioral manifestations. We used module 1, which contains 10 activities and 29 questions, for assessment because this module is largely focused on the behavioral characteristics most appropriate for characterizing the younger children found in a majority of the YouTube videos collected for this study, and also because this age group serves to benefit most from early detection and early intervention. Our aims were first to test the feasibility of answering the ADOS module 1 questions when viewing short (<10 min) unstructured videos, and second to test the accuracy of non-clinical raters to distinguish videos containing children with autism from videos of children who have no signs of autism.

## Methods

### Human Subjects

The use of videos from YouTube was approved for exemption by the Institutional Review Board at Beth Israel Deaconess Medical Center (protocol #2012P-000307), under exemption number 4 (“research involving the collection or study of existing data, documents, records, pathological specimens, or diagnostic specimens, if these sources are publicly available or if the information is recorded by the investigator in such a manner that subjects cannot be identified, directly or through identifiers linked to the subjects”) of the Code of Federal Regulations, 45 CFR 46.101(b).

### Video Selection

We searched YouTube (www.youtube.com) for videos of ASD and non-ASD children using the following keywords in the video title or description: autism, ASD, autism spectrum disorder, Asperger’s, autistic child, autistic kid, autistic behavior, PDD-NOS, son, daughter, child, birthday party. We required each video to be under 10 minutes long, have clear audible sound quality, have continuous activity, and to contain a majority of footage on a child between the ages of 1 and 15. When age was not provided it was estimated based on a consensus from two or more investigators (pertinent to 7 of the 100 videos collected). In total, we identified 100 unique subject videos, 45 ASD and 55 non-ASD. We identified non-ASD videos as controls by matching age, gender, and race as closely as possible. Videos were labeled as ASD if the video title, video description, or meta-tag included autism, ASD, Asperger’s, or hand-flapping/stimming; otherwise, the video was labeled non-ASD. The full list of videos is provided in **Table S1**.

### Raters and ADOS Scoring

ADOS scoring instruments were purchased from Western Psychological Services (http://www.wpspublish.com/). Four non-clinical raters independently scored the 29 questions on ADOS module 1 for each video. Raters were purposely given minimal instructions to code only when the video clearly depicted a behavior and/or contained opportunities for the child to exhibit the behavior in question, and otherwise to code the behavioral item as not applicable (N/A). Two of the four raters participated in the acquisition of the home videos from YouTube, and thus were not naïve to videos titles. An ADOS-certified clinical practitioner performed a blinded evaluation of a random subset of 22 videos (13 ASD and 8 non-ASD) that we used as a clinical standard for comparison to outcomes from the four non-clinical raters. We calculated the typical ADOS score as indicated in the exam instructions for the ADOS algorithm and followed the convention of converting all scores of three to two [19]. Videos were labeled ASD if the score was ≥7 and otherwise labeled as non-ASD. Any item that was scored N/A was removed from scoring and statistical analysis.

### Statistical Analysis

We computed intra-class correlations across pairs of raters for the communication and social domains and overall for the ADOS module 1. We computed the mean item-level agreement across all raters and compared the mean item-level agreement between the four non-clinical raters to the clinical rater. We calculated the accuracy, sensitivity and specificity for classifications only if the majority of raters agreed (3 out of 4). For videos where there was no majority (2 out 4) we did not make a classification and labeled it “no call.” We performed all such analyses in R [20],[21] using the “IRR” package.

## Results

We identified and analyzed 100 videos on YouTube that met our criteria for ASD (45 videos) and non-ASD (55 videos). A complete summary of video characteristics is provided in **Table 1**. The average age of children with ASD was 4.35 and 2.89 for children without an ASD. The age of the child was available in the metadata associated with the video in all but 7 of the 100 videos. For these 7, age was estimated by 2 raters independently. We found more male videos (n = 37) than female (n = 18) for ASD, which is consistent with the higher percentage of autism found in males. The most common types of video were of a child playing inside with his/her collection of toys. Nearly all videos (91%) included interaction with an adult.

**Table 1:** Characteristics of ASD and Non-ASD videos on YouTube.

Characteristic	ASD (n = 55)	Non-ASD (n = 45)
Gender^a^, % (M/F)	37/18	23/22
Age^a^, mean (range)	4.35 (1.5–15)	2.89 (0.92–6)
Race^a^		
White, %	89.1	82.2
Black, %	1.8	3.6
Other, %	9.0	13.3
Video length (min), mean (range)	3∶19 (0∶31–9∶16)	2∶37 (0∶36–6∶09)
YouTube views^b^, mean (range)	17,661 (17–96,469)	4,107,244 (146–102,871,735)
Date posted on YouTube, range	03/21/07–06/19/12	06/25/06–11/04/12
Appearance of other people^c^, %		
One person	20.0	33.3
Two or more people	18.2	17.8
Interacting with adult, %	96.4	95.6
Interacting with peer, %	16.4	17.8
Types of videos^d^, %		
Exhibiting a talent	5.5	33.3
Having a conversation	60.0	80.0
Playing	49.0	42.2
Inside	85.5	77.8
Outside	9.0	13.3
In a car	0.0	6.7
Party or birthday	1.8	8.9
Eating	10.9	22.2

a If not explicitly stated in metadata associated with the video value was estimated based on consensus of two or more investigators.

b The number of views on YouTube accessed on 12/10/13.

c Number of additional people in the video excluding the child and person recording the video.

d Videos were broadly categorized, after initial identification, to illustrate the diversity of videos evaluated.

**Figure 1:**
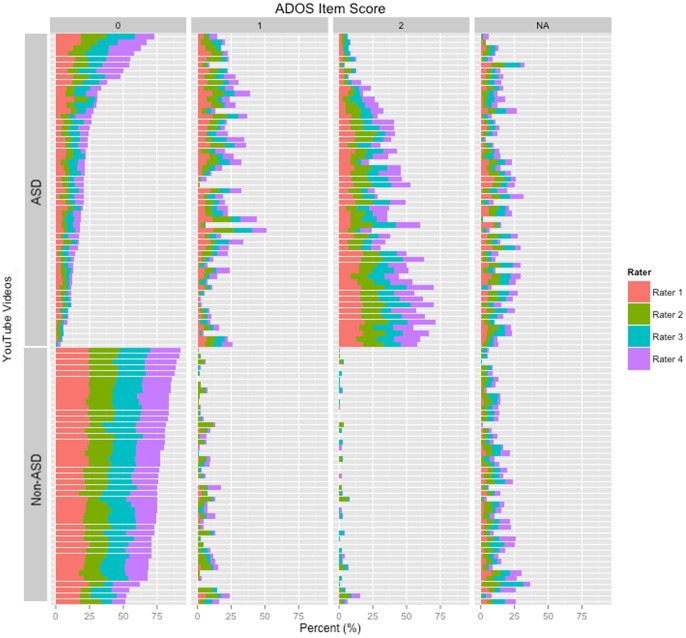
Inter-rater variability among cases previously diagnosed with autism and controls with no known diagnosis of autism. While subjectivity shifts did occur among the four independent raters, these shifts in qualitative judgment did not significantly impact the agreement among the reviewers. Both the inter-rater classification agreement (>90%) and classification accuracy (>95%) were found to be high in this analysis.

The high classification accuracy (96.8%) supported the feasibility of applying ADOS module 1 questions to videos on YouTube (**Table 2**). In addition, the high sensitivity (94.1%) and perfect specificity suggested that home video classification could yield both high positive and negative predictive value. While all behavioral items on the ADOS could be addressed by at least 1 rater in all 100 videos, 7 behaviors, including imagination and functional play, had 30% or higher N/A answer codes indicating that they were not adequately captured in the videos (**Table 3**). The intra-class correlation among raters for specific questions on ADOS module 1 were consistent with previous results [22] and ranged from 0.83–0.88. The mean rater agreement was high (73.3%) despite the diversity of environmental contexts represented in the videos. The lowest mean rater agreement was 58.1% for item B9 (“showing”) possibly due in part to the fact that it had the a relatively high frequency of N/A codes across the raters (**Table 3**). The highest mean rater agreement was 95.3% for item D3 (“self-injurious behavior”) as the children in the videos rarely exhibited this behavior. The distribution of raters’ scores for all videos is shown in **Figure 1** which provides additional insight into the classification accuracy and inter-rater agreement.

**Table 2:** Video Scoring Performance.

Characteristic	Total (n = 100)
Accuracy, %	96.8
Sensitivity, %	94.1
Specificity, %	100
No call (ASD/non-ASD)^a^	4/1
Interrater classification agreement (ASD vs. non-ASD), % mean (range)	92.2 (89–95)
Intraclass correlation across items	
Communication	0.84
Social	0.83
Total	0.88
Rater item-level mean agreement^b^, % mean (range)	73.3 (58.1–95.3)
Rater vs. Expert item-level mean agreement^c^, % mean (range)	71.3 (37.5–100)

a We required majority rater agreement (3 out of 4) for classification.

b Item-level agreement shown is the mean and range of 29 item agreements across four non-clinical raters.

c Clinical evaluation compared to four non-clinical raters for item-level agreement. All 29 items across a subset of 22 videos were considered.

**Table 3:** Scorability of each behavior across the ASD and non-ASD video collections.

	ASD		Non-ASD	
Behaviors (Code)	Code	%N/A	Code	%N/A
Anxiety (E3)	E3	0	D3	0
Facial Expressions (B3)	B3	0.0045	E2	0
Gaze to Initiate Interaction (B4)	B4	0.0045	E3	0
Repetitive Interests (D4)	D4	0.0045	A3	0.0056
Overactivity (E1)	E1	0.0045	D1	0.0056
Tantrums/Aggression (E2)	E2	0.0045	D4	0.0056
Spontaneous Expressive Language (A2)	A2	0.0091	E1	0.0056
Social Overtures (B12)	B12	0.0091	A1	0.01
Complex Mannerisms (D2)	D2	0.0091	A5	0.01
Gestures (A8)	A8	0.0182	D2	0.01
Self-Injurious Behavior (D3)	D3	0.023	B12	0.017
Eye Contact (B1)	B1	0.023	A4	0.02
Sensory Interest (D1)	D1	0.023	A8	0.028
Shared Enjoyment (B5)	B5	0.045	B1	0.028
Intonation (A3)	A3	0.086	B4	0.028
Responsive Smile (B2)	B2	0.1	A2	0.033
Idiosyncratic Use of Words (A5)	A5	0.12	B5	0.039
Pointing (A7)	A7	0.14	B3	0.044
Initiation of Joint Attention (B10)	B10	0.24	A6	0.061
Response to Name (B6)	B6	0.24	B2	0.14
Requesting (B7)	B7	0.27	B7	0.17
Showing (B9)	B9	0.29	B6	0.22
Spontaneous Expressive Language (A1)	A1	0.3	A7	0.3
Functional Play (C1)	C1	0.35	B11	0.34
Giving (B8)	B8	0.38	B10	0.35
Use of Other’s Body to Communicate (A6)	A6	0.44	B9	0.38
Echolalia (A4)	A4	0.49	C1	0.57
Imagination/Creativity (C2)	C2	0.60	B8	0.65
Social Overtures (B11)	B11	0.62	C2	0.79

The frequency of N/A (not applicable) answer codes per video over all video raters is listed in descending order for both video collections. The lowest values correspond to the most readily scored behaviors. A large majority of items were readily detectable and resulted in only a small fraction of N/As.

Because two of the raters helped to locate videos appropriate for this study, they were not always naïve to the diagnoses. To address this possible bias, we recruited a research reliable and professionally trained ADOS clinical practitioner (blinded to the diagnosis) to score 22 randomly selected videos to ensure the non-clinical raters were not unduly influenced by their prior knowledge [23] or lack of clinical experience. The raters agreed with classification provided by the clinical expert in all but 3 of the 22 randomly selected videos. In each of these 3 cases, the classification provided by the clinician did not agree with the self-reported diagnosis of ASD. The mean agreement between each non-clinical and clinical rater was 71.3%, which was consistent with the mean among non-clinical raters (73.3%) indicating that prior knowledge did not obviously bias the non-clinical rater’s scoring.

## Conclusions

The absence of any reliable molecular, neurological or physical features to characterize ASD means the best-estimate behavioral diagnosis is still the gold standard. For this reason, previous studies focused on identifying a core set of primary deficits from home videos for early age detection, to allow behavioral intervention at key stages in child development. Here, we take a different approach and apply standard diagnostic questions from ADOS module 1 to a diverse collection of videos of children in a non-clinical environment using non-clinical raters. Our results show high classification accuracy and inter-rater reliability and together demonstrate that the ADOS module 1 questions can be used by non-clinicians on unstructured videos to effectively distinguish behavioral differences among children with and without ASD.

While not all questions on the ADOS were expected to be relevant to the YouTube videos, we did find that a majority of the questions could be applied. In particular, questions regarding vocalization, use of words or phrases, unusual eye contact, responsive social smile, and repetitive interests or behaviors were the most relevant to the broad assortment of videos. Adding even a limited set of criteria to meet when capturing home videos for prospective studies may increase the likelihood that the less detectable behaviors identified in this study are captured. We observed the length of time to screen a video was never more than one minute over the length of the video and that the average assessment times were ∼4 min for ASD and ∼3∶30 min for non-ASD. These results could have important implications for faster screening approaches in the future.

There are important limitations to this proof-of-concept study. First, because we did not contact the individuals, we cannot confirm the diagnosis, gender, age, and race. Second, although by design, our raters were not trained clinicians, were not certified as research-reliable to perform ADOS and were not professionally qualified to confirm a diagnosis. Third, we applied ADOS module 1 to all the videos despite the typical recommendation that it be applied to children with single word vocabulary and mental age less than 3 years of age. However, the high agreement among the non-clinical raters, as well as the agreement of the raters to a research-reliable ADOS practitioner suggests that these limitations did not heavily bias the results.

In conclusion, we demonstrate the feasibility of applying a standard diagnostic questions to ASD and non-ASD videos from YouTube. Our results show that despite the short length (<4 minutes), home-quality, and diversity of scenarios captured within the videos it is possible to achieve good inter-rater reliability and high classification accuracy, sensitivity, and specificity. Our results also indicate that it is possible for non-clinical raters to correctly detect the presence of autism with high inter-rater reliability and >94% accuracy. These results support the potential role for short home videos in rapidly screening for a potential autism diagnosis. Based on these results, we hypothesize that it may be possible to apply other testing modules, including highly abbreviated approaches [24] to home videos to further reduce the complexity of detecting autism outside of clinical settings, without appreciable loss of accuracy in comparison to the current standard of care. Our future efforts will focus on testing this and other related hypotheses, including the potential of combining a video-based classification with brief parent-directed classification systems [25] to increase accuracy. Our results also suggest a potential larger role for public video repositories such as YouTube in the detection of other human conditions that have behavioral symptomatology.

## Supporting Information

Table S1 **Details on the 100 YouTube videos.** Forty-five autism spectrum disorder (ASD) and 55 non-ASD samples were collected in total. The Table contains the original download URL, the self-reported diagnosis, the gender and age of the subject, length of video, and the total number of YouTube views (as of 12/10/13).
(XLSX)
